# Discriminating nutritional quality of foods using the 5-Color nutrition label in the French food market: consistency with nutritional recommendations

**DOI:** 10.1186/s12937-015-0090-4

**Published:** 2015-09-28

**Authors:** Chantal Julia, Pauline Ducrot, Sandrine Péneau, Valérie Deschamps, Caroline Méjean, Léopold Fézeu, Mathilde Touvier, Serge Hercberg, Emmanuelle Kesse-Guyot

**Affiliations:** 1 Université Paris 13, Equipe de Recherche en Epidémiologie Nutritionnelle (EREN), Centre de Recherche en Epidémiologie et Statistiques, Inserm (U1153), Inra(U1125), Cnam, COMUE Sorbonne Paris Cité, 74 rue Marcel Cachin, F-93017 Bobigny, France; 2 Département de Santé Publique, Hôpital Avicenne (AP-HP), F-93017 Bobigny, France; 3 Unité de Surveillance et d’Epidémiologie Nutritionnelle (USEN), Institut de Veille Sanitaire, Bobigny, France

**Keywords:** Nutrient profiling system, Nutritional quality, Nutritional label, Discriminant performance

## Abstract

**Purpose:**

Our objectives were to assess the performance of the 5-Colour nutrition label (5-CNL) front-of-pack nutrition label based on the Food Standards Agency nutrient profiling system to discriminate nutritional quality of foods currently on the market in France and its consistency with French nutritional recommendations.

**Methods:**

Nutritional composition of 7777 foods available on the French market collected from the web-based collaborative project Open Food Facts were retrieved. Distribution of products across the 5-CNL categories according to food groups, as arranged in supermarket shelves was assessed. Distribution of similar products from different brands in the 5-CNL categories was also assessed. Discriminating performance was considered as the number of color categories present in each food group. In the case of discrepancies between the category allocation and French nutritional recommendations, adaptations of the original score were proposed.

**Results:**

Overall, the distribution of foodstuffs in the 5-CNL categories was consistent with French recommendations: 95.4 % of ‘Fruits and vegetables’, 72.5 % of ‘Cereals and potatoes’ were classified as ‘Green’ or ‘Yellow’ whereas 86.0 % of ‘Sugary snacks’ were classified as ‘Pink’ or ‘Red’. Adaptations to the original FSA score computation model were necessary for beverages, added fats and cheese in order to be consistent with French official nutritional recommendations.

**Conclusion:**

The 5-CNL label displays a high performance in discriminating nutritional quality of foods across food groups, within a food group and for similar products from different brands. Adaptations from the original model were necessary to maintain consistency with French recommendations and high performance of the system.

**Electronic supplementary material:**

The online version of this article (doi:10.1186/s12937-015-0090-4) contains supplementary material, which is available to authorized users.

## Introduction

Primary prevention of the growing burden of chronic diseases in Western countries requires multifaceted and multilevel interventions, in which nutrition may play a strategic role being a key modifiable risk factor [1–4]. Prevention programs have been developed at the state level in most Western countries and have included nutrition in their framework [5]. In France, since 2001, the National Nutrition and Health Program (Programme National Nutrition Santé, PNNS) coordinates synergistic measures, regulations and laws with the objective of improving the population’s health trough nutrition [6]. Among interventions piloted in the framework of the PNNS, nutritional recommendations pertaining to food groups consumption (e.g. ‘Five fruits and vegetables a day’, ‘Limit consumption of high fat, sweet and salty products’) are relayed by regular national multimedia campaigns and broadly disseminated through national food based dietary guidelines and food guides [7, 8].

**Fig. 1:**
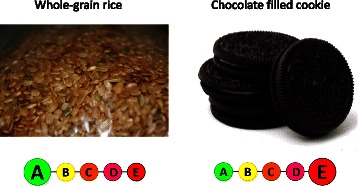
Examples of foods for which consumption is recommended and for which consumption should be limited and corresponding labelling

Beside these population-wide disseminated recommendations, recent propositions in public health nutrition in France have put forward the use of a front-of-pack nutrition label on foodstuffs, as a complementary public health tool. This label would summarize the nutritional quality of the food or beverage [9], based on the Food Standards Agency nutrient profiling system (FSA NPS score, named FSA score throughout the manuscript) [10–12]. The proposed format for the label would include five color-coded categories of nutritional quality (the 5-CNL), and presented in the form of a chain of five discs of the different colors (Green/yellow/orange/pink/red), with a larger disc representing the nutritional quality of the product (see Additional file 1: Table S1 and Fig. 1). Corresponding letters from A to E would be added in each disc to improve its readability. A/Green labelled foods correspond to foods which consumption is recommended, whereas E/Red labelled foods correspond to foods which consumption should be limited. The objective of this label would be to help consumers making healthier food choices at the point of purchase.

**Fig. 2:**
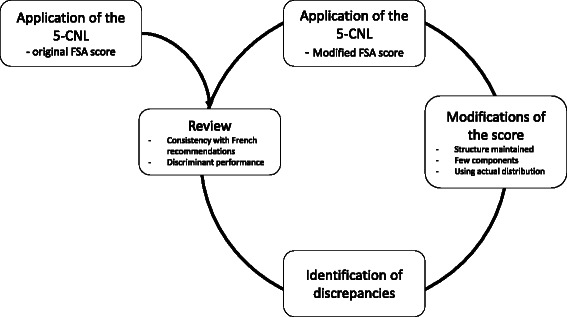
Diagram of the methods used to investigate the performance of the 5-CNL in French market foods and beverages

Recent research data tends to confirm the potential of use of the FSA score for a five-category classification of foods in France [13]. However, data related to its application to the actual food supply in France is scarce. Moreover, the FSA score has been developed specifically in the British context, and in order to regulate advertising to children, and not for labelling purposes. In turn, some adjustments or modifications may be necessary for it to be consistent so that such a system is adapted for labelling and complies with the French nutritional recommendations [13]. Indeed, as the food supply and the dietary habits of the population are very different between France and the United Kingdom, nutritional recommendations vary, though marginally. Finally, in order to be efficient in a purchasing situation, the 5-CNL would need to be able to discriminate the nutritional quality of foods across food groups (e.g. fruit and vegetables should be classified with a higher nutritional quality than snacking products), within a category (e.g. among dairy desserts, yogurt should be classified with a higher nutritional quality than chocolate pudding), but also in similar products marketed by different brands (e.g. discriminating the higher nutritional quality brand among chocolate mueslis). This discriminant capacity needs to be consistent with current variety specifically in the French food market. Discriminant performance of the 5-CNL system would therefore rely on its capacity of having multiple ‘colors’ displayed at each of these three levels of detail.

Our objectives were 1) to apply the 5-CNL using the original FSA score to foods and beverages currently on the market; 2) to investigate : the consistency of the 5-CNL against French nutritional recommendations and its discriminant performance at three levels of detail: across food groups, within a food group, and for similar products from different brands; 3) to offer and discuss potential adaptations of the original model in case of discrepancy between 5-CNL allocation and recommendations and 4) to retest consistency and discriminant performance of the 5-CNL using the modified score (Fig. 2).

**Fig. 3:**
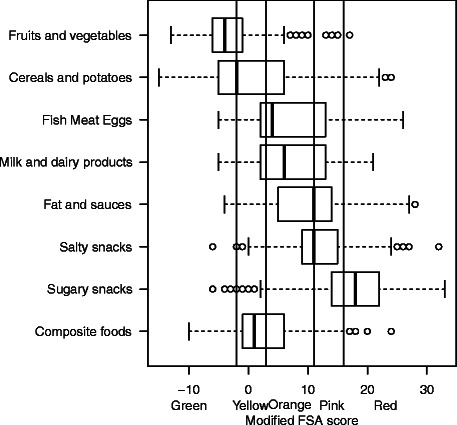
Boxplot of the distribution of food groups in the modified FSA score. Vertical lines represent the cut-offs of the 5-CNL categories. The boundary of the box nearest to the right indicates the 25th percentile, the line within the box marks the median, and the boundary of the box furthest from the right indicates the 75th percentile. Whiskers (error bars) above and below the box indicate the lower limit (25th percentile – 1.5 * (Inter-quartile range) and the upper limit (75th percentile + 1.5 * (Inter-quartile range)). The circles are individual outlier points

## Material and methods

### Food composition

Food composition data was retrieved from the Open Food Facts project database (http://world.openfoodfacts.org/). Open Food Facts is a collaborative web project gathering food composition data based on labeling from all over the world. Data is collected by volunteer contributors and includes information about ingredients and nutrition facts from food products purchased in stores. The collected data is available freely as an open data source and can be downloaded for research purposes. As the items in the database are collected from stores, foods and beverages included are exclusively manufactured pre-packaged foods. The open Food Facts database contains data from national brands, store brands and discount brands. Some products may have multiple entries if they are sold in various amounts (e.g. Nutella® can be purchased in packs of 200, 440, 630, 780 or 1 kg, each appearing in the Open Food Facts database as a separate item), leading in some cases to multiple data lines. The Open Food Facts database therefore reads as supermarket referencing. Data was retrieved from the Open Food Facts database on December 2nd, 2014. Only products marketed in France were used for the analysis. Products with complete available data for the computation of the FSA score were retained for the analyses (*n* = 7777).

### Categorization of foods

Foods were categorized using a consumer’s point of view, grouping foods with similar use, as previously described [13]. Main food groups included ‘Fruit and Vegetables’, ‘Cereals and potatoes’, ‘Meat, Fish and Eggs’, ‘Dairy products and fresh desserts’, ‘Fats and sauces’, ‘Composite dishes, ‘Sugary snacks’, ‘Salty snacks’ and ‘Beverages’. Within each category, sub-categories were identified (e.g. in the ‘Cereals and potatoes’, subcategories included ‘Bread’, ‘Pasta, rice and other cereals’, ‘Legumes’, ‘Potatoes’ and ‘Breakfast cereals’).

To investigate the performance of the 5-CNL in similar products from different brands, four foods from different groups were used as case studies: 1) mashed potatoes (from the ‘Starchy foods’ group), 2) ‘madeleine’ cakes (from the ‘Sugary snacks’ group), 3) fruit-flavoured yogurts (from the ‘Dairy products’ group) and 4) fish one-dish meals. Each of these examples represents a category from nutritional recommendations groups in France [7, 8].

### Analysis

#### Original FSA score computation and labelling category allocation

For each product, the original FSA score was computed taking into account nutrient content for 100 g. The FSA score allocates positive points (0–10) for content in energy (KJ), total sugar (g), saturated fatty acids (g) and sodium (mg). Negative points (0–5) are allocated to content in fruits, vegetables and nuts (%), fibers (g) and proteins (g). Final score is based on a discrete continuous scale ranging theoretically from −15 (higher nutritional quality) to +40 (lower nutritional quality) (see Additional file 1: Table S1). For instant hot beverages, a dilution ratio of 1.5 g per 100 g with water was applied to obtain actual composition after reconstitution.

Products were then classified into five categories. The cut-offs to define the five categories of the 5-CNL were based on previous research conducted from the food composition table of the Nutrinet-Santé study [13], which contains foods usually consumed in France, as follows from higher nutritional quality to lower nutritional quality: ‘Green’ (−15 to −2), ‘Yellow’ (−1 to 3), ‘Orange’ (4 to 11), ‘Pink’ (12 to 16) and ‘Red’ (17 and above). For beverages, the original FSA score allowed only the identification of four categories [13], which were therefore not directly converted into colors, as follows : ‘Category 1’ (−15 to −1), ‘Category 2’ (0), ‘Category 3’ (1), ‘Category 4’ (2 and above).

#### Identification of discrepancies from French nutritional recommendations

Discrepancies were identified comparing for each food group its global allocation in the 5-CNL and its recommended frequency of consumption according to PNNS guidelines. Food groups which consumption should be encouraged (e.g. ‘Fruits and vegetables’ or ‘Starchy foods’) were expected to be distributed in the ‘Green’ and ‘Yellow’ categories of the 5-CNL. In turn, food groups which consumption should be limited (e.g. salty, sugary and fatty products) were expected to be distributed in the ‘Pink’ and ‘Red’ categories of the 5-CNL. The 5-CNL was also expected to discriminate between sub-groups of foods included in nutritional recommendations and other sub-groups not included but sold in the same supermarket shelf (e.g. discriminate between ‘Milk and yogurt’ which are considered as ‘dairy products’ and ‘dairy desserts and other fresh desserts’ which are not).

For some sub-groups, specific recommendations were taken into account: for fats, discrimination between vegetable and animal added fats was expected as well as some discrimination between types of cheese.

#### FSA score adaptations

Adaptations proposed maintained the structure of the score computation and proposed modifications in the least possible number of components of the score. Modification in points attribution were proposed taking into account distribution of the continuous component (e.g. modification in attribution of saturated fat points took into account distribution of saturated fat in the considered food group) and using a homogenous ascending step to attribute points (e.g. for saturated fat, 1 point would be attributed for each 4 g/100 g). Cut-offs for attribution of the 5-CNL colors were maintained as much as possible if distribution in the different colors appeared consistent. Computation is detailed in supplemental material (see Additional file 1: Table S1).

#### Statistical analysis

Distribution of foods and beverages in the different categories of the 5-CNL were computed. Coherence of the distribution with the PNNS food groups’ recommendations was assessed, and adaptations of the original score were proposed whenever a discrepancy was identified. Distribution in the modified categories of the score was then re-assessed. Ability of the 5-CNL to discriminate nutritional quality of foods and beverages was estimated by the number of available colors in each group and for similar products from different brands as a discriminant performance indicator. When three or more colors were available in a food group, the performance of the 5-CNL was considered good, in a pragmatic approach . For equivalent products from different brands, the presence of two colors was considered as satisfactory discriminant performance.

## Results

### Application of the original FSA score

The distribution of the various food groups within the 5-CNL categories (Tables 1 and 2) was on the whole consistent with French recommendations: 95.4 % of ‘Fruits and vegetables’, 72.5 % of ‘Cereals and potatoes’ were classified as ‘Green’ or ‘Yellow’ whereas 86.0 % of ‘Sugary snacks’ were classified as ‘Pink’ or ‘Red’. Within each group, differences in nutritional quality within the various sub-groups were also grasped by the 5-CNL classification, with good discriminating performance (at least three colors present) (Table 1). For example, within ‘Dairy products and fresh desserts’, ‘Milk and yogurt’ were consistently distributed in higher nutritional quality categories than ‘Dairy desserts and other fresh desserts’; similarly, within the ‘Meat, Fish and Eggs’ category, ‘Meat’ was consistently classified as higher nutritional quality than ‘Processed meat’ (Table 1).

**Table 1:** Distribution of food groups from the Open Food Facts database in the 5-CNL using the original FSA score

		Colour category of the 5-CNL										Total
		A/Green		B/Yellow		C/Orange		D/Pink		E/Red		
		Min - -2		-1 – 3		4 – 11		11 – 16		17 – Max		
Fruits and vegetables		539	(72.1 %)	174	(23.3 %)	32	(4.3 %)	3	(0.4 %)	-	-	748
	Vegetables	355	(87.7 %)	43	(10.6 %)	5	(1.2 %)	2	(0.5 %)	-	-	405
	Dried fruits	6	(18.2 %)	22	(66.7 %)	4	(12.1 %)	1	(3.0 %)	-	-	33
	Fruits	172	(94.5 %)	7	(3.8 %)	3	(1.6 %)	-	-	-	-	182
	Soups	6	(5.8 %)	80	(77.7 %)	17	(16.5 %)	-	-	-	-	103
Cereals and potatoes		690	(51.7 %)	278	(20.8 %)	261	(19.6 %)	86	(6.4 %)	20	(1.5 %)	1335
	Bread	99	(32 %)	116	(37.5 %)	77	(24.9 %)	13	(4.2 %)	4	(1.3 %)	309
	Pasta, rice and other cereals	434	(78.6 %)	96	(17.4 %)	21	(3.8 %)	1	(0.2 %)	-	-	552
	Legumes	105	(99.1 %)	-	-	1	(0.9 %)	-	-	-	-	106
	Potatoes	37	(41.1 %)	40	(44.4 %)	10	(11.1 %)	3	(3.3 %)	-	-	90
	Breakfast cereals	15	(5.4 %)	26	(9.4 %)	152	(54.7 %)	69	(24.8 %)	16	(5.8 %)	278
Fish meat eggs		40	(5.1 %)	300	(37.9 %)	213	(26.9 %)	102	(12.9 %)	136	(17.2 %)	791
	Eggs	-	-	33	(100 %)	-	-	-	-	-	-	33
	Fish and seafood	29	(9.5 %)	150	(49.0 %)	86	(28.1 %)	40	(13.1 %)	1	(0.3 %)	306
	Meat	10	(7.8 %)	65	(50.8 %)	38	(29.7 %)	11	(8.6 %)	4	(3.1 %)	128
	Offals	1	(6.7 %)	2	(13.3 %)	2	(13.3 %)	9	(60.0 %)	1	(6.7 %)	15
	Processed meat	-	-	50	(16.2 %)	87	(28.2 %)	42	(13.6 %)	130	(42.1 %)	309
Milk, dairy products and desserts		48	(5.2 %)	316	(34.1 %)	194	(20.9 %)	147	(15.8 %)	223	(24.0 %)	928
	Milk and yogurt	46	(10.9 %)	274	(64.8 %)	67	(15.8 %)	29	(6.9 %)	7	(1.7 %)	423
	Cheese	-	-	9	(3.5 %)	3	(1.2 %)	56	(22 %)	187	(73.3 %)	255
	Dairy desserts and other desserts	2	(1.5 %)	23	(16.9 %)	76	(55.9 %)	32	(23.5 %)	3	(2.2 %)	136
	Ice cream	-	-	10	(8.8 %)	48	(42.1 %)	30	(26.3 %)	26	(22.8 %)	114
Fat and sauces		10	(2.2 %)	72	(15.6 %)	88	(19.1 %)	115	(24.9 %)	176	(38.2 %)	461
	Dressings and sauces	10	(3.7 %)	71	(26.1 %)	84	(30.9 %)	73	(26.8 %)	34	(12.5 %)	272
	Fats	-	-	1	(0.5 %)	4	(2.1 %)	42	(22.2 %)	142	(75.1 %)	189
Salty snacks		14	(2.9 %)	47	(9.8 %)	216	(45.0 %)	123	(25.6 %)	80	(16.7 %)	480
	Appetizers	2	(0.6 %)	12	(3.7 %)	154	(47.7 %)	94	(29.1 %)	61	(18.9 %)	323
	Nuts	9	(15.5 %)	17	(29.3 %)	29	(50.0 %)	3	(5.2 %)	-	-	58
	Salty and fatty products	3	(3.0 %)	18	(18.2 %)	33	(33.3 %)	26	(26.3 %)	19	(19.2 %)	99
Sugary snacks		7	(0.5 %)	27	(1.8 %)	172	(11.7 %)	369	(25.2 %)	892	(60.8 %)	1467
	Biscuits and cakes	4	(0.5 %)	12	(1.5 %)	67	(8.5 %)	212	(26.8 %)	497	(62.8 %)	792
	Chocolate products	-	-	2	(0.5 %)	34	(9.0 %)	32	(8.5 %)	310	(82.0 %)	378
	Pastries	1	(1.1 %)	4	(4.6 %)	15	(17.2 %)	41	(47.1 %)	26	(29.9 %)	87
	Sweets	2	(1.0 %)	9	(4.3 %)	56	(26.7 %)	84	(40 %)	59	(28.1 %)	210
Composite foods		153	(19.8 %)	382	(49.4 %)	160	(20.7 %)	61	(7.9 %)	18	(2.3 %)	774
	One-dish meals	121	(20.4 %)	321	(54.1 %)	115	(19.4 %)	28	(4.7 %)	8	(1.3 %)	593
	Pizza pies and quiche	2	(2.6 %)	21	(27.3 %)	32	(41.6 %)	18	(23.4 %)	4	(5.2 %)	77
	Sandwich	6	(11.5 %)	14	(26.9 %)	11	(21.2 %)	15	(28.8 %)	6	(11.5 %)	52
	Side dishes	24	(46.2 %)	26	(50 %)	2	(3.8 %)	-	-	-	-	52

Data are *n*(raw %)

**Table 2:** Distribution of beverages in the 5-CNL using the original FSA score

	Categories of nutritional quality								
	Category 1		Category 2		Category 3		Category 4		
	Min - -1		0		1		2 – Max		*N*
Beverages	301	(38 %)	166	(20.9 %)	124	(15.6 %)	202	(25.5 %)	793
Water and flavoured water	-	-	20	(100 %)	-	-	-	-	20
Tea, herbal tea and coffee	-	-	55	(100 %)	-	-	-	-	55
Fruit juices	284	(99.3 %)	-	-	1	(0.3 %)	1	(0.3 %)	286
Fruit nectars	-	-	-	-	6	(17.6 %)	28	(82.4 %)	34
Fruit flavoured still drinks	15	(19.2 %)	6	(7.7 %)	30	(38.5 %)	27	(34.6 %)	78
Artificially sweetened beverages	1	(1.3 %)	71	(88.8 %)	5	(6.3 %)	3	(3.8 %)	80
Sweetened beverages	1	(0.4 %)	14	(5.8 %)	82	(34.2 %)	143	(59.6 %)	240

Data are *n*(raw %)

For similar products from different brands, at least two ‘colors’ were identified each time: mashed potatoes distributed in four colors from ‘Green’ to ‘Pink’, fruit yogurts in ‘Yellow’ and ‘Orange’, Madeleine cakes in ‘Pink’ and ‘Red’, and finally fish one-dish meals in three colors, from ‘Green’ to ‘Orange’ (Table 3).

**Table 3:** Distribution equivalent products from the Open Food Facts database in the 5-CNL using the FSA score

	Colour category of the 5-CNL					
	A/Green	B/Yellow	C/Orange	D/Pink	E/Red	Total
Madeleine cakes	-	-	-	9 (69.2 %)	4 (30.8 %)	13
Fish one-dish meals	11 (28.9 %)	25 (65.8 %)	2 (5.3 %)	-	-	38
Mashed potatoes	16 (64 %)	2 (8 %)	5 (20 %)	2 (8 %)	-	25
Fruit yogurt	-	27 (79.4 %)	7 (20.6 %)	-	-	34

Data are *n*(raw %)

### Identification of discrepancies with French nutritional recommendations

The original FSA score failed to achieve its objectives for some categories: nuts and dried fruit, beverages, cheese and added fat.

In France, nuts and dried fruits are considered as snacking products, as they are mostly consumed as appetizers. Therefore, these food groups’ consumption is not encouraged [7, 8]. The distribution of the ‘Nuts’ (15.5 % as ‘Green’) and ‘Dried Fruits’ (18.2 % as ‘Green’) subgroups within the 5-CNL for the original FSA score are therefore not consistent with recommendations (Table 1).

Moreover, the PNNS includes cheese in the ‘Dairy products’ category, considering it as a good source of calcium [14]. Therefore, the distribution of this group, with 73.3 % in ‘Red’ does not correspond to this recommendation (Table 1).

The PNNS recommends to privilege vegetable added fats to animal added fats, and to encourage diversity in the types of fats used [7, 8]. However, the distribution in the 5-CNL colors using the original FSA score does not allow grasping differences in types of fats, as 75.1 % of added fats are classified in the ‘Red’ category, whether vegetable or animal (Table 1).

Finally, the only recommended beverage in the PNNS is water (‘drink water liberally according to thirst’). Sweetened beverages’ consumption (including fruit juices) should be limited, and whenever possible, artificially sweetened beverages should be preferred to regular sweetened options [7, 8]. However, distribution of beverages in the original FSA score does not reflect these considerations. Indeed, fruit juices have lower score than water, and artificially sweetened beverages have the same score as water (Table 2). Moreover, variability of score for beverages is very low.

### Adaptations to the original score

For each of the food groups for which the 5-CNL allocation displayed discrepancies with French nutritional recommendations, adaptations of the original score were proposed.

#### Nuts and dried fruit

The Fruits, vegetables and nuts component of the original FSA score includes content in fruits, dried fruits, vegetables, legumes and nuts. Indeed, in the UK nuts are considered as a good source of proteins and dried fruits as equivalent to fruits. The original score was adapted to exclude dried fruits and nuts from the computation of the fruit and vegetables component of the FSA score. After modification, the ‘Nuts’ and ‘Dried fruit’ subgroups are classified mainly as ‘Orange’ (62.1 and 72.7 %, respectively Table 4 and Fig. 3).

**Table 4:** Distribution of food groups from the Open Food Facts database in the 5-CNL using the modified FSA score

		Colour category of the 5-CNL										Total
		A/Green		B/Yellow		C/Orange		D/Pink		E/Red		
		Min - -2		-1–3		4–11		11–16		17–Max		
Fruits and vegetables		533	(71.3 %)	158	(21.1 %)	49	(6.6 %)	6	(0.8 %)	2	(0.3 %)	748
	Vegetables	355	(87.7 %)	43	(10.6 %)	5	(1.2 %)	2	(0.5 %)	-	-	405
	Dried fruits^a^	-	-	6	(18.2 %)	24	(72.7 %)	2	(6.1 %)	1	(3.0 %)	33
	Fruits	172	(94.5 %)	7	(3.8 %)	3	(1.6 %)	-	-	-	-	182
	Soups	6	(5.8 %)	80	(77.7 %)	17	(16.5 %)	-	-	-	-	103
Cereals and potatoes		690	(51.7 %)	278	(20.8 %)	261	(19.6 %)	86	(6.4 %)	20	(1.5 %)	1335
	Bread	99	(32 %)	116	(37.5 %)	77	(24.9 %)	13	(4.2 %)	4	(1.3 %)	309
	Pasta, rice and other cereals	434	(78.6 %)	96	(17.4 %)	21	(3.8 %)	1	(0.2 %)	-	-	552
	Legumes	105	(99.1 %)	-	-	1	(0.9 %)	-	-	-	-	106
	Potatoes	37	(41.1 %)	40	(44.4 %)	10	(11.1 %)	3	(3.3 %)	-	-	90
	Breakfast cereals	15	(5.4 %)	26	(9.4 %)	152	(54.7 %)	69	(24.8 %)	16	(5.8 %)	278
Fish Meat Eggs		40	(5.1 %)	300	(37.9 %)	213	(26.9 %)	102	(12.9 %)	136	(17.2 %)	791
	Eggs	-	-	33	(100 %)	-	-	-	-	-	-	33
	Fish and seafood	29	(9.5 %)	150	(49.0 %)	86	(28.1 %)	40	(13.1 %)	1	(0.3 %)	306
	Meat	10	(7.8 %)	65	(50.8 %)	38	(29.7 %)	11	(8.6 %)	4	(3.1 %)	128
	Offals	1	(6.7 %)	2	(13.3 %)	2	(13.3 %)	9	(60.0 %)	1	(6.7 %)	15
	Processed meat	-	-	50	(16.2 %)	87	(28.2 %)	42	(13.6 %)	130	(42.1 %)	309
Milk, dairy products and desserts		48	(5.2 %)	316	(34.1 %)	245	(26.4 %)	249	(26.8 %)	70	(7.5 %)	928
	Milk and yogurt	46	(10.9 %)	274	(64.8 %)	67	(15.8 %)	29	(6.9 %)	7	(1.7 %)	423
	Cheese^a^	-	-	9	(3.5 %)	54	(21.2 %)	158	(62.0 %)	34	(13.3 %)	255
	Dairy desserts and other desserts	2	(1.5 %)	23	(16.9 %)	76	(55.9 %)	32	(23.5 %)	3	(2.2 %)	136
	Ice cream	-	-	10	(8.8 %)	48	(42.1 %)	30	(26.3 %)	26	(22.8 %)	114
Fat and sauces		10	(2.2 %)	74	(16.1 %)	156	(33.8 %)	144	(31.2 %)	77	(16.7 %)	461
	Dressings and sauces	10	(3.7 %)	71	(26.1 %)	84	(30.9 %)	73	(26.8 %)	34	(12.5 %)	272
	Fats^a^	-	-	3	(1.6 %)	72	(38.1 %)	71	(37.6 %)	43	(22.8 %)	189
Salty snacks		5	(1.0 %)	39	(8.1 %)	223	(46.5 %)	130	(27.1 %)	83	(17.3 %)	480
	Appetizers	2	(0.6 %)	12	(3.7 %)	154	(47.7 %)	94	(29.1 %)	61	(18.9 %)	323
	Nuts^a^	-	-	9	(15.5 %)	36	(62.1 %)	10	(17.2 %)	3	(5.2 %)	58
	Salty and fatty products	3	(3.0 %)	18	(18.2 %)	33	(33.3 %)	26	(26.3 %)	19	(19.2 %)	99
Sugary snacks		7	(0.5 %)	27	(1.8 %)	172	(11.7 %)	369	(25.2 %)	892	(60.8 %)	1467
	Biscuits and cakes	4	(0.5 %)	12	(1.5 %)	67	(8.5 %)	212	(26.8 %)	497	(62.8 %)	792
	Chocolate products	-	-	2	(0.5 %)	34	(9.0 %)	32	(8.5 %)	310	(82.0 %)	378
	Pastries	1	(1.1 %)	4	(4.6 %)	15	(17.2 %)	41	(47.1 %)	26	(29.9 %)	87
	Sweets	2	(1.0 %)	9	(4.3 %)	56	(26.7 %)	84	(40.0 %)	59	(28.1 %)	210
Composite foods		153	(19.8 %)	382	(49.4 %)	160	(20.7 %)	61	(7.9 %)	18	(2.3 %)	774
	One-dish meals	121	(20.4 %)	321	(54.1 %)	115	(19.4 %)	28	(4.7 %)	8	(1.3 %)	593
	Pizza pies and quiche	2	(2.6 %)	21	(27.3 %)	32	(41.6 %)	18	(23.4 %)	4	(5.2 %)	77
	Sandwich	6	(11.5 %)	14	(26.9 %)	11	(21.2 %)	15	(28.8 %)	6	(11.5 %)	52
	Side dishes	24	(46.2 %)	26	(50.0 %)	2	(3.8 %)	-	-	-	-	52

Data are *n*(raw %) Foods with ^a^ have a modified distribution from the original score

#### Cheese

**Fig. 4:**
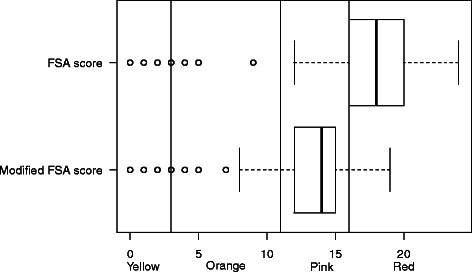
Boxplot of the distribution of cheese in the original and modified FSA score. Vertical lines represent the cut-offs of the 5-CNL categories. The boundary of the box nearest to the right indicates the 25th percentile, the line within the box marks the median, and the boundary of the box furthest from the right indicates the 75th percentile. Whiskers (error bars) above and below the box indicate the lower limit (25th percentile – 1.5 * (Inter-quartile range) and the upper limit (75th percentile + 1.5 * (Inter-quartile range)). The circles are individual outlier points

During the development of the model for the FSA score by the Food Standards Agency, content in calcium was initially considered as one of the components, and later discarded, as taking into account protein content was highly correlated to it [11]. However, the model was later amended so that products high in sugar, salt, saturated fat and energy would not be classified as ‘healthier’ through a high content in proteins [11]. Score computation therefore does not take into account proteins if the total A points is higher than 11 (see Additional file 1: Table S1). Such a modification leads to discard the protein content – and therefore the closely-related calcium content – in cheese, which usually have a total A points >11, due to high content in saturated fat (73.3 % of cheese is classified as ‘Red’, Table 1). We modified the original score for cheese, so that protein content would be used in the computation, whatever the initial total of A points of the product. Cheese distribution after modification is still in lower nutritional categories than ‘Milk and yogurt’, but is distributed across ‘Orange’, ‘Pink’ and ‘Red’ categories (21.2, 62.0 and 13.3 % respectively, Table 4 and Fig. 4).

#### Added fats

**Fig. 5:**
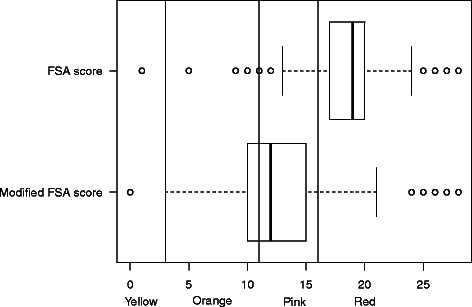
Boxplot of the distribution of added fats in the original and modified FSA score. Vertical lines represent the cut-offs of the 5-CNL categories. The boundary of the box nearest to the right indicates the 25th percentile, the line within the box marks the median, and the boundary of the box furthest from the right indicates the 75th percentile. Whiskers (error bars) above and below the box indicate the lower limit (25th percentile – 1.5 * (Inter-quartile range) and the upper limit (75th percentile + 1.5 * (Inter-quartile range)). The circles are individual outlier points

The fact that the maximum number of points for saturated fat is achieved with 10 g/100 g can explain the lack of discrimination between types of added fats observed using the original FSA score. However, saturated fat content is differential across types of added fats, from 80 g/100 g for butter to 20–30 g/100 g for margarines and vegetable fats. Modifying point allocation for saturated fat would allow to redistribute added fats within multiple categories of the 5-CNL and to discriminate between animal and vegetable fats. Given the distribution of saturated fats in added fats, an ascending step of one point for each 4 g of saturated/ 100 g was used (see Additional file 1: Table S1). Such modification led to a distribution of fats with 38.1 % in ‘Orange’, mostly light margarines, 37.6 % in ‘Pink’, mostly vegetable oils and regular margarine, 22.8 % in ‘Red’, butter and palm oil exclusively (Table 4 and Fig. 5).

#### Beverages

**Fig. 6:**
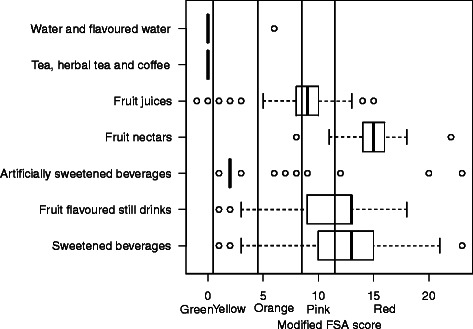
Boxplot of the distribution of beverages in the modified FSA score. Vertical lines represent the cut-offs of the 5-CNL categories. The boundary of the box nearest to the right indicates the 25th percentile, the line within the box marks the median, and the boundary of the box furthest from the right indicates the 75th percentile. Whiskers (error bars) above and below the box indicate the lower limit (25th percentile – 1.5 * (Inter-quartile range) and the upper limit (75th percentile + 1.5 * (Inter-quartile range)). The circles are individual outlier points

To achieve the 5-CNL goals, the FSA score energy and sugar components of the score were modified taking into account the distribution of energy and sugars in beverages. An ascending step of 30 KJ was used for energy and an ascending step of 1.5 g/100 ml for sugars. Moreover, for artificially sweetened beverages, at least one point for sugar was allocated, even though the content was null, in order to maintain a positive score. Finally, cut-offs for the different categories were modified, as follows: ≤0 ‘Green’, 1–4 ‘Yellow’, 5–8 ‘Orange’, 9–11 ‘Pink’, ≥12, ‘Red’. After modification, water and tea, coffee and herbal tea were classified as ‘Green’, artificially sweetened beverages as ‘Yellow’, fruit juices as ‘Orange’ and ‘Pink’ and most sweetened beverages as ‘Red’ (Table 5 and Fig. 6).

**Table 5:** Distribution of beverages from the Open Food Facts database in the 5-CNL using the modified FSA score

		Colour category of the 5-CNL										Total
		A/Green		B/Yellow		C/Orange		D/Pink		E/Red		
		Min–0		1–4		5–8		9–11		12–Max		
Beverages^a^		76	(9.6 %)	95	(12 %)	106	(13.4 %)	251	(31.7 %)	265	(33.4 %)	793
	Water and flavoured water^a^	19	(95 %)	-	-	1	(5 %)	-	-	-	-	20
	Tea, herbal tea and coffee^a^	55	(100 %)	-	-	-	-	-	-	-	-	55
	Fruit juices^a^	2	(0.7 %)	7	(2.4 %)	72	(25.2 %)	178	(62.2 %)	27	(9.4 %)	286
	Fruit nectars^a^	-	-	-	-	2	(5.9 %)	1	(2.9 %)	31	(91.2 %)	34
	Fruit flavoured still drinks^a^	-	-	10	(12.8 %)	3	(3.8 %)	15	(19.2 %)	50	(64.1 %)	78
	Artificially sweetened beverages^a^	-	-	69	(86.3 %)	6	(7.5 %)	1	(1.3 %)	4	(5.0 %)	80
	Sweetened beverages^a^	-	-	9	(3.8 %)	22	(9.2 %)	56	(23.3 %)	153	(63.8 %)	240

Data are *n*(raw %) Foods with a ^a^ have a modified distribution from the original score

## Discussion

Our study shows that the 5-CNL, based on an amended FSA score allows to discriminating across food groups, within food groups and for similar products across different brands, for foods products and beverages currently available on the French market. Overall, classification of products according to the 5-CNL was consistent with nutritional recommendations: foods which consumption is recommended (e.g. fruits and vegetables) were better classified than foods which consumption should be limited (e.g. sugary and salty snacks). Within a food group, the same discrimination was observed, as foods lower in salt, sugar and fat were better classified. For some food groups, adaptations to the original score increased its discriminant performance and its consistency with French nutritional recommendations. The adaptations proposed to the original score to be consistent with official nutritional recommendations included only four food groups: nuts and dried fruits, beverages, cheese and added fats. These adaptations proceed from two main reasons: first, the FSA score was developed to regulate advertising to children, with a binary outcome (advertising authorized/forbidden). It was therefore not necessarily adapted for some food groups that are not targeted to children. Second, the adaptations account for the differences between French and British nutritional recommendations, which are each adapted to their specific populations and their dietary habits.

Compared to the Australian front-of-package label ‘Health Star Rating system’, which also relies on a modified version of the FSA score, adaptations of the original model were conducted on very similar food groups (namely beverages, added fats and spreads and dairy products), confirming that the discrepancies identified for the original model applied to the French context in our study were consistent with other national nutritional programs [15]. Additionally, the results observed in our study are consistent with those investigating the consistency of the FSA score, using nutritional composition data from a database used for scientific purpose, confirming the stability of results using two different sources of data [13].

Front-of-pack labels currently in use in Europe include nutrient-specific labels, either factual (e.g. Guidelines Daily Amounts (GDAs), displaying the relative contribution for a portion size in energy, fat, saturated fat, sugar and sodium of a given product [16]) or with a color-coding (e.g. ‘Traffic lights’, displaying ‘green’ ‘amber’ or ‘red’ dots for content per 100 g in the same nutrients [17]) and single indicators of nutritional quality, using nutrient profiling systems (the Danish green ‘Keyhole’ [18] or the Dutch ‘Choices’ [19]). However, numeric information only (as in the ‘GDA’ system) appears to have a much lower impact on food choices than more salient systems [20, 21]. Moreover, single indicators of nutritional quality seem to be better understood and used than nutrient-specific labels. However, nutrient profiling systems currently in use as a basis for front-of-package labeling in Europe exclusively involve simple labels, the label being present only for foods meeting specific requirements. Such dichotomization of foods in ‘healthier’ and ‘less healthy’ categories could however lead to dichotomized thinking, promoting the contention that foods are either ‘all good’ or ‘all bad’ [22]. Moreover, research data tends to support graded nutritional labels, considered as easy to identify and allowing more clearly the comparison of the nutritional quality of foods, as they offer a good balance of simplicity and salience [23]. The 5-CNL approach, combining a single indicator of the global nutritional quality of the food, the use of color-coding for higher readability [24] and the inclusion of five graded categories rather than a binary assessment can therefore be considered as an innovative format. Moreover, it relies on the FSA nutrient profiling system, which is currently the most validated [10, 25, 26] and in line with current EU regulations concerning nutritional values mentioned on the back of the package [27].

Nutritional recommendations to modify dietary behaviors are considered to refer either to a promotion of ‘displacement’ of consumptions from one group to another (e.g. ‘Five fruits and vegetables a day) or of ‘substitution’ within one food group from less healthy foods to more healthy alternatives (e.g. ‘Limit consumption of high fat, sweet and salty products’) [26]. On the other hand, front-of-pack labeling based on nutrient profiling systems can be divided in across-the-board (the computation is the same whatever the food group considered) and category-specific labels (computation depends on the food group considered) [25, 26]. While ‘across-the-board’ labeling is thought to support the ‘displacement’ theory, ‘category-specific’ labels would support the ‘substitution’ theory [26]. The investigation of individual diets using the FSA score of foods consumed showed that both ‘displacement’ and ‘substitution’ strategies were involved in the comparison of healthier vs. less healthy diets [26]. Authors concluded that efficient nutrient profiling systems would require a category-specific approach, but with very few categories [26]. Our results tend to show that the use of a modified FSA score associated with the 5-CNL, while being ‘across-the-board’ from most food items, responds to these imperative and would support both ‘displacement’ and ‘substitution’ strategies, as nutritional quality across food groups, but also within food groups is consistently discriminated.

Our study is subject to some limitations. First, though the Open Food Facts database collects data from products currently on the market, we were not able to analyze the representativeness of the sample of foods retrieved, either in terms of number of products or market share. However, our purpose was not to be exhaustive, but rather to test the performance of the 5-CNL in real-life situations, for which the Open Food Facts database is sufficiently large to give a consistent evaluation. Moreover, consistent with current EU regulations on food labeling [27], the 5-CNL would be used as a voluntary initiative by manufacturers and stakeholders [9]. Its endorsement by the French government would guarantee a standardization of front-of-pack labeling existing in France, but not its universal application. Caution is therefore warranted as to the efficiency of such a labeling scheme, as recent research data suggest that rather than processing the information delivered by nutritional labels, consumers tend to consider the mere existence of a label as a sign of healthiness of the food [28]. However, the formats used in this study were all nutrient-specific labels (GDA and Traffic Light labels). Single indicators of nutritional quality (such as the 5-CNL) are thought to imply less consumer cognitive processing and therefore be more effective to help consumers analyzing the quality of a product in real-life time-constrained situations [29–32]. Future studies should therefore investigate the use and effectiveness of the 5-CNL in purchase situations in France in order to assess its potential impact in terms of diet, and ultimately, health gains.

## Conclusion

The 5-CNL appears as a useful tool which allows discriminating nutritional quality of foods at various levels of detail in foods marketed in France. Modifications of the original FSA model to ensure a higher consistency with French nutritional recommendations increased the discriminant performance of the 5-CNL. The 5-CNL would allow consumers making healthier choices at the point of purchase.

## Additiona file

Additiona file 1: Table S1. Modified FSA score computation. (DOCX 23 kb)

## Abbreviations

FSA: Food Standards Agency
NPS: Nutrient profiling system
PNNS: Programme National Nutrition Santé
5-CNL: Five-Color Nutrition label
