# Supplementary material for: Discriminating nutritional quality of foods using the 5-Color nutrition label in the French food market: consistency with nutritional recommendations
Source: Nutr J. 2015 Sep 28;14:100. doi: 10.1186/s12937-015-0090-4 (PMC4587869; doi:10.1186/s12937-015-0090-4)
Supplement: Additiona file 1: Table S1. — Modified FSA score computation. (DOCX 23 kb) [file 12937_2015_90_MOESM1_ESM.docx]

**Online supplemental Table 1 Modified FSA score computation**

Points are allocated according to the nutrient content for 100g of foods or beverages.

Points are allocated for ‘Negative’ nutrients (A points) and can be balanced according to ‘Positive’ nutrients (C points).

**A points**

Total A points = (points for energy) + (points for saturated fat) + (points for total sugar) + (points for sodium)

| \| ***Points*** \| **Energy (kJ)** \| **Saturated Fat (g)** \| **Total Sugars (g)** \| **Sodium (mg)** \| \| --- \| --- \| --- \| --- \| --- \| \| 0 \| ≤ 335 \| ≤ 1 \| ≤ 4.5 \| ≤ 90 \| \| 1 \| >335 \| >1 \| >4.5 \| >90 \| \| 2 \| >670 \| >2 \| >9 \| >180 \| \| 3 \| >1005 \| >3 \| >13.5 \| >270 \| \| 4 \| >1340 \| >4 \| >18 \| >360 \| \| 5 \| >1675 \| >5 \| >22.5 \| >450 \| \| 6 \| >2010 \| >6 \| >27 \| >540 \| \| 7 \| >2345 \| >7 \| >31 \| >630 \| \| 8 \| >2680 \| >8 \| >36 \| >720 \| \| 9 \| >3015 \| >9 \| >40 \| >810 \| \| 10 \| >3350 \| >10 \| >45 \| >900 \|   **C points**  Total C points = (points for fruits and vegetables) + (points for fibers) + (points for proteins)   \| ***Points*** \| **Fruits, Vegetables (%)** \| **Fiber (g) *** \| **Protein (g)** \| \| --- \| --- \| --- \| --- \| \| 0 \| ≤ 40 \| ≤ 0.7 \| ≤ 1.6 \| \| 1 \| >40 \| >0.7 \| >1.6 \| \| 2 \| >60 \| >1.4 \| >3.2 \| \| 3 \| - \| >2.1 \| >4.8 \| \| 4 \| - \| >2.8 \| >6.4 \| \| 5 \| >80 \| >3.5 \| >8.0 \| |
| --- | --- | --- | --- | --- | --- | --- | --- | --- | --- | --- | --- | --- | --- | --- | --- | --- | --- | --- | --- | --- | --- | --- | --- | --- | --- | --- | --- | --- | --- | --- | --- | --- | --- | --- | --- | --- | --- | --- | --- | --- | --- | --- | --- | --- | --- | --- | --- | --- | --- | --- | --- | --- | --- | --- | --- | --- | --- | --- | --- | --- | --- | --- | --- | --- | --- | --- | --- | --- | --- | --- | --- | --- | --- | --- | --- | --- | --- | --- | --- | --- | --- | --- | --- | --- | --- | --- | --- | --- |

*FSA score allocates different thresholds for fibers, depending on the measurement method used. We used NSP cut-offs to compute fibers score.

**Overall score computation**

- If Total A points <11, then FSA score =Total A points – Total C points
- If Total A points ≥11,
  - If points for fruits and vegetables =5, then FSA score =Total A points – Total C points
  - Else if points for fruits and vegetables <5, then FSA score = Total A points – (points for fiber + points for fruits and vegetables).

The percentage of fruits and vegetables was calculated taking into account fruits, legumes and vegetables as defined in the PNNS (the French nutritional and health policy). Tubers, oleaginous fruits, dried fruits and olives are therefore not considered in this computation.

Exceptions were made for cheese, fat, and drinks to better rank them according to their nutrient profile, consistently with nutritional recommendations:

**Score computation for cheese**

For cheese, the score takes in account the protein content, whether the A score reaches 11 or not, i.e.: FSA score =Total A points – Total C points

**Score computation for fat**

For fat, the grid for points attribution regarding saturated fat has a four-points homogenous ascending step, as shown thereafter:

| ***Points*** | **Saturated Fat (g)** |
| --- | --- |
| 0 | < 6 |
| 1 | ≥ 6 |
| 2 | ≥ 10 |
| 3 | ≥ 14 |
| 4 | ≥ 18 |
| 5 | ≥ 22 |
| 6 | ≥ 26 |
| 7 | ≥ 30 |
| 8 | ≥ 34 |
| 9 | ≥ 38 |
| 10 | ≥ 42 |

**Score computation for drinks**

For drinks, the grids for points attribution regarding energy and total sugars were modified. The attribution of points for sugars takes into account the presence of sweeteners, in which case the grid maintains the total sugar score to 1 (instead of 0).

| ***Points*** | **Energy (kJ)** | **Total Sugar (g)** |
| --- | --- | --- |
| 0 | ≤0 | ≤0 except if sweeteners |
| 1 | ≤30 | ≤1.5 or if sweeteners |
| 2 | ≤60 | ≤3 |
| 3 | ≤90 | ≤4.5 |
| 4 | ≤120 | ≤6 |
| 5 | ≤150 | ≤7.5 |
| 6 | ≤180 | ≤9 |
| 7 | ≤210 | ≤10.5 |
| 8 | ≤240 | ≤12 |
| 9 | ≤270 | ≤13.5 |
| 10 | Above | Above |

Milk and vegetable milk are not concerned by this exception. Their scores are computed using the overall score computation system.

**5 Color Nutrition Label attribution**

**Foods**

| Category 1 | Min — -2 | Green |
| --- | --- | --- |
| Category 2 | -1 — 3 | Yellow |
| Category 3 | 4 — 11 | Orange |
| Category 4 | 12 — 16 | Pink |
| Category 5 | 17 — Max | Red |

**Beverages**

| Category 1 | Min — 0 | Green |
| --- | --- | --- |
| Category 2 | 1 — 4 | Yellow |
| Category 3 | 5 — 8 | Orange |
| Category 4 | 9 — 11 | Pink |
| Category 5 | 12 — Max | Red |
